# Effects of Energy-Restricted Feeding during Rearing on the Performance, Uniformity, and Development of Rugao Layer Breeders at the Initiation of the Laying Period

**DOI:** 10.3390/ani11082222

**Published:** 2021-07-28

**Authors:** Jian Lu, Liang Qu, Yongfeng Li, Meng Ma, Manman Shen, Xingguo Wang, Jun Guo, Yuping Hu, Taocun Dou, Zhi Yang, Kehua Wang

**Affiliations:** 1 Poultry Institute, Chinese Academy of Agricultural Sciences, Yangzhou 225125, China; lujian1617@163.com (J.L.); liyf0120@163.com (Y.L.); meng-2005@163.com (M.M.); yzwangxingguo@163.com (X.W.); smileleaf@yeah.net (J.G.); 15371333966@163.com (Y.H.); 2 College of Animal Science and Technology, Nanjing Agricultural University, Nanjing 210095, China; 3 Key Laboratory for Poultry Genetics and Breeding of Jiangsu province, Yangzhou 225125, China; shenman2005@163.com (M.S.); yzdtc@126.com (T.D.); 4 Joint International Research Laboratory of Agriculture and Agri-Product Safety of Ministry of Education of China, Yangzhou University, Yangzhou 225009, China; zhiyang@yzu.edu.cn

**Keywords:** layer breeder, energy-restricted feeding, performance, uniformity, development

## Abstract

**Simple Summary:**

Three major factors affecting productive performance of laying hens are BW, flock uniformity, and GIT development at the initiation of the laying period. Various feeding management practices to restrict feed intake of broiler breeders during the rearing phase to optimize BW for reproductive performance can improve BW uniformity. Hence, the feed restriction methods used for broiler breeders might be used to improve flock uniformity and the GIT development of laying hens. The objective of the study was to investigate the effects of energy-restricted feeding and switching to ad libitum feeding on the performance, uniformity, and development of Rugao layer breeders at the initiation of the laying period. Moderate energy restriction from 8 to 18 weeks of age and switching to ad libitum feeding can stimulate the development of the GIT and improve BW uniformity of layer breeders. Improved ECR was observed overall in the experiment. In addition, the BW of layer breeders recovered after the pullets were switched to ad libitum feeding for 3 weeks. These results provide a theoretical basis for the application of energy-restricted feeding in young layer breeders, which may have important practical importance for layer breeders because a better rearing cycle can be advantageous to production performance.

**Abstract:**

The aim of this study was to assess the effects of energy-restricted feeding during rearing on the performance, uniformity, and development of layer breeders at the initiation of the laying period. A total of 2400 8-week-old Rugao layer breeders were randomly assigned to one of five groups (480 pullets per group) with eight replicates and were fed one of five diets that were nutritionally equal with the exception of apparent metabolizable energy corrected for nitrogen (AME_n_) content (2850, 2750, 2650, 2550, and 2450 kcal AME_n_/kg) from 8 to 18 weeks of age. The daily amount of feed was restricted to the absolute quantity of the diet consumed by laying hens fed 2850 kcal AME_n_ per kg diet ad libitum (control). From 18 to 21 weeks of age, all hens were fed a basal diet ad libitum. The body weight (BW) of the laying pullets decreased linearly with increasing energy restriction (*p* < 0.001) but recovered within 3 weeks of ad libitum feeding (*p* = 0.290). A gradual increase in the degree of energy restriction resulted in a gradual decrease in average daily weight gain (ADG) and a gradual increase in the feed conversion ratio (FCR) and energy conversion ratio (ECR) from 8 to 18 weeks of age (*p* < 0.001, *p* < 0.001, *p* = 0.008). In contrast, the ADG and ADFI (*p* < 0.001, *p* < 0.001) gradually increased, while the FCR and ECR (*p* < 0.001, *p* < 0.001) gradually improved from 18 to 21 weeks of age. From 8 to 21 weeks of age, ECR improved (*p* = 0.005) with an increasing degree of energy restriction. The energy-restricted feeding for 6 weeks to the end of the trial improved BW uniformity (*p* < 0.05). The relative length and circumference of tarsus (*p* < 0.001, *p* < 0.001), and the relative weights and lengths of the small intestine, duodenum, jejunum, ileum, and caeca increased linearly (*p* < 0.001, *p* = 0.012, *p* < 0.007, *p* = 0.012, *p* = 0.040; *p* < 0.001, *p* = 0.003, *p* = 0.032, *p* = 0.029, *p* = 0.040) with increasing energy restriction at 18 weeks of age. After switching to *ad libitum* feeding for 3 weeks, the relative weights and lengths of the small intestine, duodenum, and jejunum of laying pullets increased linearly with increasing energy restriction (*p* < 0.001, *p* = 0.016, *p* = 0.011; *p* = 0.009, *p* = 0.028, *p* = 0.032). In conclusion, moderate energy restriction (85.97%, 2450 vs. 2850 kcal AME_n_/kg) from 8 to 18 weeks of age and switching to ad libitum feeding from 18 to 21 weeks of age can be used to improve BW uniformity and stimulate the development of the duodenum and jejunum of native layer breeders at the initiation of the laying period without compromising BW.

## 1. Introduction

The rearing period is one of the most important stages in the life of a laying hen and any mistakes made during this period cannot be corrected or adjusted during the subsequent laying period [1]. The development of size, skeleton, and gastrointestinal tract (GIT) mainly occur during rearing period [1]. Thus, three major factors that affect the productive performance of laying hens are body weight (BW), flock uniformity, and GIT development at the onset of the egg-laying cycle [2]. Consequently, it is essential to meet the target BW, achieve high flock uniformity, and improve the GIT development for laying pullets before being delivered into the laying house.

Poor flock uniformity before the start of the laying period prevents maximum production because of suboptimal performance of both overweight and underweight hens [1]. Low, as opposed to excess, BW of laying hens at the initiation of the laying period results in fewer and smaller eggs produced during the whole laying cycle [3,4,5]. In addition, a well-developed GIT is expected to adapt to the consumption of increased amounts of feed in the subsequent laying period. However, relatively few studies have focused on the BW, flock uniformity, and GIT development of laying hens during the rearing period [6,7,8,9,10]. Various feeding management practices to restrict feed intake of broiler breeders during the rearing phase to optimize BW for reproductive performance can improve BW uniformity [11,12,13]. Hence, the feed restriction methods used for broiler breeders might improve flock uniformity of laying hens in the early rearing period and ad libitum feeding in the late rearing period might improve GIT development and satisfy the nutrient requirement for the onset of sexual maturity and persistent egg production [14] because of the higher feed and nutrient intake required for compensatory growth.

The Rugao laying hen was approved as the national cultivated laying hen breed of China by the National Examination and Approval Committee of Domestic Animal and Poultry Breeds in 2009. It is typically used as the female parent of Suqin blue-eggshell chickens, which was approved as a crossbreeding system in 2013. The average BW of a Rugao laying hen is about 1.1 and 1.7 kg at 18 and 52 weeks of age, respectively [15]. Consequently, the Rugao laying hen can be used as a typical model of the certified crossbreeding systems in China.

The hypothesis tested in this study was that energy-restricted feeding in the earlier rearing period might improve flock uniformity of young layer breeders, but adversely affect BW. Also, ad libitum feeding can allow layer breeders to achieve the target weight and improve development of the GIT because of the higher feed and nutrient intake required for compensatory growth. The objective of the study was to investigate the effects of energy-restricted feeding and switching to ad libitum feeding on the performance, uniformity, and development of Rugao layer breeders at the initiation of the laying period.

## 2. Materials and Methods

### 2.1. Study Design, Birds, and Diets

A total of 2400, 8-week-old, Rugao layer breeders were randomly assigned to one of five groups (480 pullets per group) with eight replicates of 60 pullets each. From 8 to 18 weeks of age, the laying hens were fed one of five diets that were nutritionally equal except for apparent metabolizable energy corrected for nitrogen (AMEn) content. The calculated AME_n_ values were 2850, 2750, 2650, 2550, and 2450 kcal/kg, respectively (Table 1). Feed was provided ad libitum for the laying hens provided with 2850 kcal AME_n_ per kg diet (control). The amount of feed offered to the laying hens in the other groups was restricted to the absolute quantity of the diet consumed by the pullets in the control group. From 18 to 21 weeks of age, all experimental laying hens were switched to a basal diet (Table 2) formulated to meet the National Research Council [16] recommendations for laying hens. Water and feed were provided ad libitum.

Samples of diets were ground to pass through a 40-mesh sieve and immediately frozen and stored at −20 °C for further analysis. The moisture contents of the diets were determined by oven-drying [17]. Gross energy was determined with an adiabatic bomb calorimeter (Model 1356; Parr Instrument Company, Moline, IL, USA). Calcium content was determined by atomic absorption spectrophotometry (Beijing Beifen-Ruili Analytical Instrument (Group) Co., Ltd., Yangzhou, China; 927.02) [17]. Phosphorus content was determined photometrically in orthophosphate from filtered ash solutions with the vanado-molybdate method [17]. The total nitrogen content of the samples was determined with the micro-Kjeldahl method [17]. Crude protein (CP) was calculated as nitrogen × 6.25. The amino acid content was assayed according to the method described by Wang et al. [18] with an ion-exchange high-performance liquid chromatography system (Waters Corporation, Wilford, MA, USA) in accordance with AOAC method 994.12 (sulfur and regular) [17], with postcolumn o-phthalaldehyde derivatization and fluorescence detection following acid hydrolysis. Duplicate samples of the diets (5 mg) were hydrolyzed in 1000 µL of 6 M HCl containing 0.1% phenol for 24 h at 110 ± 2 °C in vacuum-sealed glass tubes. The contents of glycine and tryptophan, which are destroyed by acid hydrolysis, were not determined.

### 2.2. Husbandry

This trial was carried out at the Poultry Institute of the Chinese Academy of Agricultural Sciences (Yangzhou City, Jiangsu, China) from July to October 2019. From 8 to 18 weeks of age, each replicate of 60 hens was randomly assigned to 20 cages of three hens each. From 18 to 21 weeks of age, all hens were transferred to a laying house and caged individually at a constant temperature of 24 ± 3 °C and relative humidity of 65–75%. Light exposure was limited to 8 h from 8 to 18 weeks of age and then increased by 1 h per week until the end of the experiment. One cage remained empty, and a chipboard was inserted into the feeders between the different replicate cages to prevent hens in one replicate from eating the feed of another. All animal handing protocols were approved by the Animal Care and Use Committee of the Poultry Institute (SYXK(Su)IACUC 2012-0029). Cage-side observations, which included recording any changes in clinical condition or behavior, were made at least twice per day throughout the study period.

### 2.3. Sample Collection and Analytical Determination

#### 2.3.1. Performance

Individual BW and feed intake by replicate were measured on a weekly basis, and when mortality was recorded, the pullets were weighed. The average daily weight gain (ADG), average daily feed intake (ADFI), and feed conversion ratio (FCR) were determined by period and cumulatively from these data. FCR is expressed as grams of feed consumed per gram of BW gain. In addition, the average daily energy intake (ADEI), expressed as kcal AMEn consumed per day, and the energy conversion ratio (ECR), expressed as kcal calculated AMEn per g BW gain, were also estimated by period and cumulatively.

#### 2.3.2. Uniformity

The pullets were weighed individually at weekly intervals and BW uniformity was determined by replicates, as described by Peak et al. [19] and Guzmán et al. [8]. Briefly, the coefficient of variation (CV) was used as an indirect measurement of BW uniformity among the laying pullets. The coefficient of variation was calculated using the following formula:

CV = (standard deviation/average BW) × 100%.

#### 2.3.3. Development 

At 18 and 21 weeks of age, the tarsus length of all birds was measured with a digital caliper and the tarsus circumference was measured (at the midpoint of the bone) three times with a cotton thread, which was measured with a ruler with a precision of 1 mm. The relative values were calculated as a ratio to live BW.

At 18 and 21 weeks of age, after fasting for 12 h, four birds from each replicate were killed by exsanguination and subjected to full postmortem examinations. The breast meat (including the pectoralis major and minor muscles), thigh meat (including the thigh and drumstick), abdominal fat, head, wing, foot, heart, liver, spleen, lung, and kidney were weighed (paired organs were weighed together), and the ratios of weight to live BW were calculated.

Afterward, the full proventriculus and gizzard were carefully excised, emptied of any digesta, cleaned, dried with desiccant paper, and weighed. Then, the number of papillae in the proventriculus was recorded. The relative values were calculated as the ratio to live BW. The length of each intestinal segment was measured using a flexible tape on a glass surface to prevent inadvertent stretching, as described by Lu et al. [20]. The lengths (±0.01 cm) of the duodenum (from the pyloric junction to the most distal point of insertion of the duodenal mesentery), jejunum (from the most distal point of insertion of the duodenal mesentery to the junction with Meckel’s diverticulum), ileum (from the junction with Meckel’s diverticulum to the ileocecal junction), cecum, and rectum were determined. After division and freeing of each intestinal segment, separating all connective tissue and fat, and removing the content by flushing with ice-cold saline, the empty weight (±0.01 g) and length of each segment were determined along with those of the proventriculus and gizzard. The relative values were calculated as a ratio of live BW.

### 2.4. Statistical Analysis

All data analyses were conducted using SPSS for Windows, version 16.0 (SPSS Inc., Chicago, IL, USA). One-way analysis of variance followed by Duncan’s multiple comparison test was used to identify differences in means among treatments. Regression curve estimation was also used to determine linear and quadratic responses of hens to different energy restrictions. Differences were reported where *p* ≤ 0.05.

## 3. Results

### 3.1. BW

**Figure 1:**
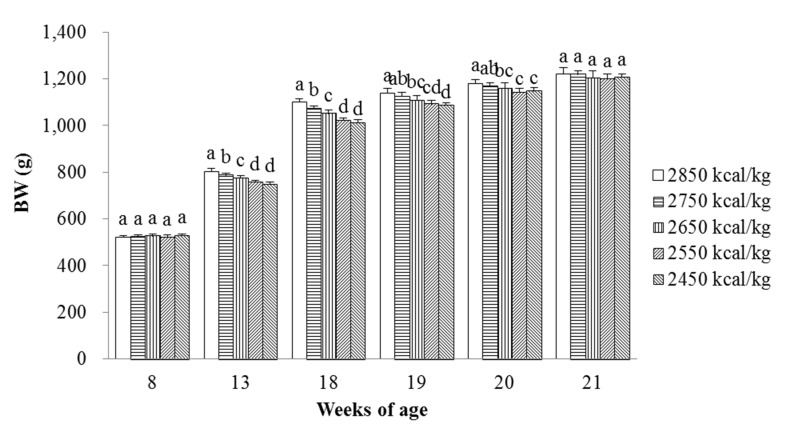
Effects of energy-restricted feeding and switching to ad libitum feeding on the BW of pullets from 8 to 21 weeks of age. Values are means of eight replicates per dietary treatment. Columns with different superscripts (a–d) are significantly different at *p* < 0.05.

The effects of energy-restricted feeding and switching to ad libitum feeding on the BW of the laying hens are presented in Figure 1. There was no significant difference in initial BW. The BW of laying hens at 13 and 18 weeks of age (after 5 and 10 weeks of energy-restricted feeding) decreased linearly with increasing energy restriction (*p* < 0.001, *p* < 0.001), and similar significant differences in BW were also observed after the switch to ad libitum feeding for the first and second weeks (*p* < 0.001, *p* = 0.001). There was no significant difference in BW among the groups after 3 weeks of the switch to ad libitum feeding (*p* = 0.290).

### 3.2. Visual Observations and Performance

**Figure 2:**
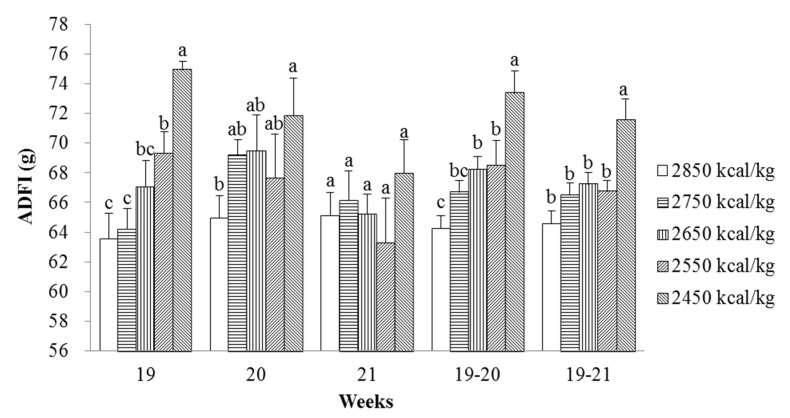
Effects of energy-restricted feeding and switching to ad libitum feeding on ADFI (g/bird per day) of the pullets from 18 to 21 weeks of age. Values are means of eight replicates per dietary treatment. Columns with different superscripts (a–c) are significantly different at *p* < 0.05.

No treatment-related adverse clinical signs were observed. As shown in Table 3, the degree of energy restriction gradually increased from 8 to 18 weeks of age along with a gradual decrease in ADG and a gradual increase in the FCR from 8 to 13 (*p* < 0.001, *p* < 0.001), 13 to 18 (*p <* 0.001, *p <* 0.001), and 8 to 18 weeks of age (*p* < 0.001, *p* < 0.001). In contrast, a gradual increase in ADG and ADFI (*p* < 0.001, *p* < 0.001), and a gradual improvement in the FCR (*p* < 0.001) were observed from 18 to 21 weeks of age. The ADFI of laying pullets in the more severe energy restriction groups increased linearly after the switch to ad libitum feeding for the first and second weeks (*p* < 0.001, *p* = 0.039), then gradually decreased to a normal level relative to that of the control group in the third week (*p* = 0.437, Figure 2). From 8 to 21 weeks of age, the ADFI and FCR increased linearly (*p* < 0.001, *p* < 0.001) with increasing energy restriction from 8 to 18 weeks of age, whereas no differences were detected in ADG (*p* = 0.180) and mortality (*p* = 0.972).

### 3.3. ADEI and ECR

The ADEI decreased linearly from 8 to 13 (*p* < 0.001), 13 to 18 (*p* < 0.001), and 8 to 18 weeks of age (*p* < 0.001), and the ECR increased linearly from 8 to 13 (*p* < 0.001) and 8 to 18 weeks of age (*p* = 0.008) with increasing degree of energy restriction (Table 4). In contrast, the ADEI from 18 to 21 weeks of age increased linearly (*p* < 0.001), whereas the ECR improved linearly (*p* < 0.001) as the energy restriction levels increased from 8 to 18 weeks of age. From 8 to 21 weeks of age, a gradual increase in the degree of energy restriction from 8 to 18 weeks of age was associated with a gradual decrease in ADEI and a gradual improvement in ECR (*p* < 0.001, *p* = 0.005).

### 3.4. BW Uniformity

**Figure 3:**
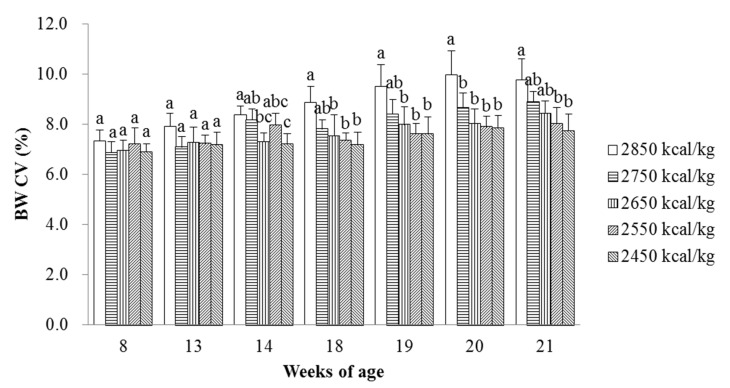
Effects of energy-restricted feeding and switching to ad libitum feeding on BW coefficient of variation (CV) of the pullets from 8 to 21 weeks of age. Values are means of eight replicates per dietary treatment. Columns with different superscripts (a–c) are significantly different at *p* < 0.05.

The effects of energy-restricted feeding and switching to ad libitum feeding on the BW uniformity of the laying hens are presented in Figure 3. No statistically significant changes (*p* > 0.05) were observed in the BW uniformity of pullets administered different energy restriction levels until 14 weeks of age. The BW uniformity was significantly improved from energy-restricted feeding for 6 weeks to the end of the trial (*p* < 0.05).

### 3.5. Tarsus Development

After 10 weeks of energy-restricted feeding, no statistically significant changes were observed in absolute tarsus length (*p* > 0.05, Table 5), whereas the absolute tarsus circumference decreased linearly (*p* = 0.005), and the relative length and circumference of the tarsus increased linearly (*p* < 0.001, *p* < 0.001) with increasing energy restriction from 8 to 18 weeks of age. There was no statistically significant change in tarsus development after the laying pullets were switched to ad libitum feeding for 3 weeks.

### 3.6. Carcass and Visceral Organ Development

After 10 weeks of energy-restricted feeding, no statistically significant changes were observed in the carcass and visceral organ development (*p* > 0.05, Table 6), except for the abdominal fat and liver (at 18 weeks of age). More severe restriction of dietary energy from 8 to 18 weeks of age decreased the abdominal fat index and increased the liver index linearly (*p* = 0.010, *p* = 0.030). However, there were no statistically significant changes in the carcass and visceral organ development after the laying pullets were switched to ad libitum feeding for 3 weeks.

### 3.7. GIT Development

The relative wet weights of the small intestine, duodenum, jejunum, ileum, and caeca increased linearly (*p* < 0.001, *p* = 0.012, *p* = 0.007, *p* = 0.012, *p* = 0.040), while the proventriculus papilla (PP) index tended to increase linearly (*p* = 0.066) with increasing energy restriction at 18 weeks of age (Table 7). In addition, the laying pullets assigned to gradually increasing restriction had significantly heavier relative weights of the small intestine, duodenum, and jejunum at 21 weeks of age (*p* < 0.001, *p* = 0.016, *p* = 0.011; Table 7).

Significant increases in the relative lengths of the small intestine, duodenum, jejunum, ileum, and caeca (*p* < 0.001, *p* = 0.003, *p* = 0.032, *p* = 0.029, *p* = 0.040) occurred with an increase in the degree of energy restriction at 18 weeks of age (Table 8). In addition, the relative lengths of the small intestine, duodenum, and jejunum increased linearly with increasing energy restriction after the laying pullets were switched to ad libitum feeding for 3 weeks (*p* = 0.009, *p* = 0.028, *p* = 0.032; Table 8).

## 4. Discussion

The diets of each treatment group were nutritionally equal with the exception of energy concentration. The ADFI of laying pullets among dietary groups was the same from 8 to 18 weeks of age. Consequently, the ADEI increased or decreased with the dietary energy concentration. Briefly, the difference in energy-restricted feeding among the treatments in this trial was reflected as the difference in ADEI from 8 to 18 weeks of age.

In the present study, the BW of laying pullets decreased after energy-restricted feeding and lasted until switching to ad libitum feeding for 2 weeks, whereas BW recovered within 3 weeks after the laying pullets were switched to ad libitum feeding for 3 weeks. Relatively few studies have investigated feed restriction in laying pullets in the rearing period. Recent studies have indicated that the BW of broilers in feed-restricted groups tended to be lower directly after the period of restriction and the differences in BW disappeared within 1 week after the feed restriction period for feed-restricted broilers during the second or third week of life [21]. In addition, Butzen et al. [22] reported that broilers provided with 80% of the ad libitum intake from 8 to 16 days of age do not differ in BW at 35 days of age as compared with ad libitum-fed broilers. Urdaneta-Rincon and Leeson [23] have shown that feed restriction of 10% from day 14 to 17, 20, 23, 26, or 29 decreases the BW of male broilers at day 35, but not at day 42 or 49. Novel et al. [24] have shown that feed restriction of 50%, but not 25%, from 14 to 21 days of age decreased BW of both male and female broilers at day 42. The results of the present study may be explained by the energy intake in the earlier rearing period (from 8 to 18 weeks of age) being so low that even though the nutrient intake (feed intake) increased after the laying pullets were switched to ad libitum feeding for 1 or 2 weeks, the differences in the BW of laying pullets between the energy-restricted feeding and control groups remained. In addition, the immediate decrease in the BW of laying pullets in response to energy-restricted feeding indicated a mismatch between energy intake and growth requirements. After switching to ad libitum feeding, the ADFI, ADEI, and ADG of laying pullets increased linearly as the energy restriction increased from 8 to 18 weeks of age and the feed efficiency might be improved. Therefore, laying pullets displayed prompt compensatory growth following the period of energy restriction, and the BW was recovered at 3 weeks after the restriction period. The data reported in this paper suggest that young laying pullets have ability to regulate BW when switching from energy-restricted feeding to ad libitum feeding. These results are consistent with our predictions that energy-restricted feeding in earlier rearing periods would adversely affect BW, but the laying pullets were able to catch up to the target BW after the switch to ad libitum feeding because of the improved feed efficiency associated with compensatory growth.

In this study, a gradual increase in the degree of energy restriction from 8 to 18 weeks of age resulted in a gradual decrease in the ADG and gradual increases in the FCR and ECR from 8 to 18 weeks of age. In contrast, the ADG and ADFI gradually increased, and the FCR and ECR gradually improved from 18 to 21 weeks of age. From 8 to 21 weeks of age, the ECR improved linearly as the energy restriction increased from 8 to 18 weeks of age, but the FCR increased linearly. The results of this experiment were consistent with most previous studies in broilers [21,25]. Hornick et al. [25] reported that broilers normally experience a period of rapid compensatory growth following a period of feed restriction. Moreover, van der Klein et al. [21] reported that feeding both 80% and 70% of ad libitum consumption in week 2, and 85% and 80% of ad libitum consumption in week 3 broilers resulted in a greater ADG in the week after feed restriction than that in the ad libitum-fed group. However, Butzen et al. [22] reported that 80% of ad libitum consumption from 8 to 16 days of age had no effect the FCR of broilers until 35 days of age. Novel et al. [24] also reported that restriction of both 50% and 25% from 14 to 21 days of age had no effect on the FCR of male and female broilers until 42 days of age. In contrast, Urdaneta-Rincon and Leeson [23] reported that feed restriction of 10% from day 14 to 17, 20, 23, 26, or 29 improved the FCR of male broilers overall: the longer the feed restriction, the lower the cumulative FCR until day 42. Palo et al. [26] also reported that 70% of ad libitum consumption from day 7 to 14 reduced the overall FCR from 1.87 to 1.80 until day 48 in broilers. The extent to which laying hens show compensatory growth is dependent on many factors, including the environment, period, and method of the applied restriction, as well as strain and sex. As expected, in the current study, laying pullets in the most severe energy restriction group achieved the greatest compensatory growth, which may have resulted from increased feed intake. The present study demonstrated that metabolic rate of laying pullets may be adjusted to become thriftier after reaching a certain level of energy restriction [12], and a certain threshold level of energy restriction may be necessary to adjust the metabolic rate of laying pullets in order to realize the advantages of compensatory growth. Apparently, the degree of energy restriction from 8 to 18 weeks of age was severe enough to result in beneficial compensatory growth. Therefore, energy-restricted feeding from 8 to 18 weeks of age and switching to ad libitum feeding can improve the ECR of laying pullets from 18 to 21 and 8 to 21 weeks of age.

High flock uniformity around the time of sexual maturation is expected because laying pullets that are uniform in BW should be more uniform in the onset of production, whereas poor uniformity is difficult to improve by simply adjusting feed and lighting. In the current study, energy-restricted feeding improved BW uniformity, and better flock uniformity was observed following energy-restricted feeding for 6 weeks to the end of the trial (switching to ad libitum feeding for 3 weeks). Implementation of these commercial strategies can improve flock uniformity of laying hens. Available data on the influence of energy-restricted feeding during the rearing phases on flock uniformity of laying pullets are very limited for comparison with the results of the present study. In broiler breeders, qualitative diet dilution and skip-a-day management did little to increase flock uniformity relative to controls during the most intense period of feed restriction (7 to 19 weeks of age), but skip-a-day treatment, compared with qualitative dilution treatment, improved flock uniformity at 22 weeks of age [11]. de Beer and Coon [27] reported an increase in flock uniformity of broiler breeders by skip-a-day treatments versus everyday limited feeding. However, van der Klein et al. [21] reported that feed restriction at weeks 2 and 3 resulted in no difference in the BW uniformity of broilers compared with that of the ad libitum-fed group. In this trial, BW uniformity improved linearly with increasing energy restriction, a result potentially attributable to a decrease in metabolic stress. Considerable evidence suggests that the maintenance energy requirement fluctuates with age, feed intake, environmental temperature, and the degree of feed restriction [12,28]. The degree of energy restriction from 8 to 18 weeks of age was severe enough to improve the BW uniformity. In contrast, factors, such as the type and age of the birds, flock management, nutrient density of the feed, quantity of feed provided, frequency and timing of feed delivery, stocking pressure, feeder design, and feeder space, all affect flock uniformity.

In the current study, a gradual increase in the degree of energy restriction was associated with a gradual increase in the relative tarsus length and circumference, and a gradual decrease in the absolute tarsus circumference at 18 weeks of age, but no significant difference in tarsus development in pullets was observed at 21 weeks of age (after the switch to ad libitum feeding for 3 weeks). The tarsus length and circumference have been used to predict the growth and future size of birds [6,7,29,30]. However, available data on the influence of energy-restricted feeding during the rearing phases on these variables in laying pullets are very limited. Zuidhof [31] indicated that the carcass yield increases as the BW of birds increases because of differences in the allometric growth of tissues and body organs. Therefore, the data presented in this trial indicate that energy-restricted feeding from 8 to 18 weeks of age can improve tarsus development of pullets, but the advantage in tarsus development is not long-lasting and gradually disappears because of prompt compensatory growth. The pullets under more severe energy restriction treatment might have lower maintenance requirements and, therefore, could direct more nutrients toward growth and development in the subsequent ad libitum feeding period.

In the current study, after 10 weeks of energy-restricted feeding, no statistically significant changes were observed in the carcass and visceral organ development with the exceptions of abdominal fat and liver (at 18 weeks of age). In addition, no statistically significant changes in the carcass and visceral organ development were observed after the switch to ad libitum feeding for 3 weeks. The results of this experiment are consistent with those of many studies of broilers. For example, Urdaneta-Rincon and Leeson [23] reported that feed restriction of 10% from day 14 to 17, 20, 23, 26, or 29 had no effect on abdominal fat pads of male broilers at day 42 or 49. In addition, Butzen et al. [22] reported that 80% of ad libitum consumption from 8 to 16 days of age had no effect on breast muscle weight or fat content of male broilers at 35 and 42 days of age. Velleman et al. [32] also reported that 20% feed restriction during the first week after hatching increases fat deposition in the breast muscle, but 20% feed restriction during the second week of life had no effect on breast muscle weight of male broilers at day 42. In addition, van der Klein et al. [21], when comparing ad libitum feeding with 90, 80, or 70% restricted broilers, observed that broilers fed 70% of ad libitum consumption during the second week achieved full compensatory growth within 1 week after the restriction period. The degree of energy restriction from 8 to 18 weeks of age was not severe enough to result in a significant decrease in all carcasses and visceral organs. The liver is the main organ for fat metabolism; therefore, increased liver weight might be an indicator of increased hepatic activity. During energy-restricted feeding, more severe restriction of dietary energy might increase the mobilization of energy stored in tissues to satisfy energy needs, including the fatty acids made available from adipose tissue, glucose released from liver glycogen stores, and further energy provided by catabolism of muscle protein through gluconeogenesis. Therefore, the liver index increased, and the abdominal fat index decreased linearly at 18 weeks of age with increasing degree of energy restriction, indicating that energy-restricted feeding might alter metabolism of laying pullets. In addition, a period of 3 weeks of ad libitum feeding after energy restriction is sufficient to allow the abdominal fat and liver of birds to recover, and the data reported in this paper suggest that the laying pullets achieved complete compensatory growth within 3 weeks after the restriction period.

The relative weights and lengths of all gut segments, with the exception of the rectum, increased and the PP index tended to increase linearly with increasing energy restriction at 18 weeks of age. The number of papilla-like projections in the proventriculus is a measure of the proventriculus trait because each papilla can digest protein via the secretion of gastric juices [33]. After the switch to ad libitum feeding for 3 weeks, the relative weights and lengths of the small intestine, duodenum, and jejunum of laying pullets also increased linearly with increasing energy restriction. Saldaña et al. [6] found that the energy concentration in the diet does not affect the relative weights of the full proventriculus and gizzard or the relative lengths of the small intestine and caeca at 5, 10, and 17 weeks of age (ad libitum feeding). In contrast, Frikha et al. [2] observed that an increase in the AMEn concentration of the diet reduced the relative weights of all segments of the GIT at 45 days of age and the relative weight of the gizzard at 120 days of age of laying pullets (2735, 2880 to 3025 kcal/kg; ad libitum feeding). In addition, van der Klein et al. [21] observed that feeding 80% of ad libitum consumption during the third week of life decreases the GIT weight in female broilers at day 35, but not males. The differences in the ages of the birds, feed intake, nutrient density of feed, and restriction levels might explain the discrepancy among some of these results. The diets in each treatment were nutritionally equal, with the exception of AME_n_ content, and the ADFI of laying pullets among the dietary groups remained the same from 8 to 18 weeks of age. Consequently, energy-restricted feeding from 8 to 18 weeks of age might stimulate the development and physiology of the GIT, thus improving nutrient digestibility to meet maintenance requirements. After the switch to a basal diet, the ADFI of laying pullets in the more severe energy restriction groups increased linearly after the switch for the first and second weeks, then gradually decreased to a normal level relative to that of the control group in the 3rd week, resulting in greater relative weights and lengths of the small intestine, duodenum, and jejunum. However, the differences in ADFI among the treatments were probably too small to produce any marked difference in the sizes of the proventriculus, gizzard, and other gut segments of the laying pullets. The potential beneficial effects of energy-restricted feeding from 8 to 18 weeks of age on the size and development of the GIT might not be long-lasting and may gradually disappear after feeding a common commercial layer diet.

## 5. Conclusions 

The findings of the present study revealed that moderate energy restriction (down to 85.97% of ad libitum intake, 2450 vs. 2850 kcal AME_n_/kg) from 8 to 18 weeks of age and switching to ad libitum feeding can stimulate the development of the GIT and improve BW uniformity of layer breeders, as flock uniformity was improved by energy-restricted feeding for 6 weeks to the end of the trial (diet-switching to the basal diet for 3 weeks). Improved ECR was observed overall in the experiment. In addition, the BW of layer breeders recovered after the pullets were switched to ad libitum feeding for 3 weeks. Furthermore, these results provide a theoretical basis for the application of energy-restricted feeding in young laying pullets, which may have important practical importance for layer breeders because a better rearing cycle can be advantageous to production performance.

## Figures and Tables

**Table 1:** Ingredients and nutrient levels of experimental diet provided from 8 to 18 weeks of age. ^1.^

AME_n_, kcal/kg	Control	Energy-Restricted Feeding			
	2850	2750	2650	2550	2450
Ingredient (%)					
Corn	71.95	68.47	64.99	61.50	58.02
Soybean meal	21.80	22.17	23.03	23.65	24.27
Quartz sand	2	2	2	2	2
Limestone	1.56	1.55	1.55	1.55	1.55
Zeolite powder	0.67	3.78	6.40	9.27	12.12
Calcium hydrogen phosphate	0.87	0.88	0.88	0.88	0.89
Sodium chloride	0.35	0.35	0.35	0.35	0.35
50% Choline chloride	0.20	0.20	0.20	0.20	0.20
*DL*-Met	0.10	0.10	0.10	0.10	0.10
Vitamin and trace mineral premix ^2^	0.50	0.50	0.50	0.50	0.50
Nutrient levels (calculated)					
AME_n_ (kcal/kg)	2850	2750	2650	2550	2450
Crude protein (%)	15.50	15.50	15.50	15.50	15.50
Digestible amino acid (%)					
Lysine	0.67	0.66	0.67	0.66	0.66
Methionine	0.32	0.32	0.32	0.32	0.32
Methionine + Cystine	0.54	0.53	0.53	0.54	0.53
Arginine	0.99	0.99	1.00	1.00	1.01
Threonine	0.45	0.45	0.45	0.46	0.46
Calcium (%)	0.88	0.88	0.88	0.88	0.88
Total phosphorus (%)	0.56	0.55	0.55	0.54	0.54
Digestible phosphorus (%)	0.38	0.38	0.38	0.38	0.38
Nutrient levels (measured)					
DM (%)	92.2	91.8	93.1	92.6	92.3
Gross energy (kcal/kg)	3977	3812	3769	3603	3562
Crude protein (%)	15.57	15.55	15.63	15.61	15.58
Total amino acid (%)					
Lysine	0.77	0.78	0.79	0.80	0.77
Methionine	0.34	0.33	0.34	0.34	0.35
Methionine + Cystine	0.65	0.65	0.65	0.65	0.65
Arginine	1.09	1.10	1.08	1.07	1.09
Threonine	0.54	0.56	0.55	0.57	0.55
Calcium (%)	0.90	0.90	0.86	0.88	0.91
Total phosphorus (%)	0.57	0.53	0.56	0.56	0.55

^1^ Values are expressed on an air-dry basis. ^2^ Premix includes (per kilogram of diet): vitamin A, 8800 IU; vitamin D_3_, 3300 IU; vitamin E, 60 IU; cobalamin, 23 μg; riboflavin, 5.5 mg; niacin, 30 mg; pantothenic acid, 8 mg; choline, 500 mg; menadione, 1.2 mg; folic acid, 0.9 mg; pyridoxine, 1.2 mg; thiamine, 1.7 mg; biotin, 55 μg; manganese, 90 mg; zinc, 86 mg; iron, 55 mg; copper, 5.5 mg; iodine, 1.6 mg; selenium, 0.3 mg. AME_n_, apparent metabolizable energy corrected for nitrogen.

**Table 2:** Ingredients and nutrient levels of the experimental diet provided from 18 to 21 weeks of age. ^1.^

	Ad Libitum Feeding
Ingredient (%)	
Corn	66.00
Soybean meal	25.00
Wheat	1.67
Shell powder	3.33
Limestone	1.33
Zeolite powder	0.60
Calcium hydrogen phosphate	1.00
Sodium chloride	0.30
50% Choline chloride	0.17
*DL*-Met	0.10
Vitamin and trace mineral premix ^2^	0.50
Nutrient levels (calculated)	
AME_n_ (kcal/kg)	2750
Crude protein (%)	17.00
Digestible amino acid (%)	
Lysine	0.73
Methionine	0.34
Methionine + Cystine	0.56
Arginine	1.08
Threonine	0.49
Calcium (%)	2.00
Total phosphorus (%)	0.60
Digestible phosphorus (%)	0.40
Nutrient levels (measured)	
DM (%)	92.7
Gross energy (kcal/kg)	3904
Crude protein (%)	16.93
Total amino acid (%)	
Lysine	0.83
Methionine	0..37
Methionine + Cystine	0.67
Arginine	1.15
Threonine	0.62
Calcium (%)	1.98
Total phosphorus (%)	0.62

^1^ Values are expressed on an air-dry basis. ^2^ Premix includes (per kilogram of diet): vitamin A, 8800 IU; vitamin D_3_, 3300 IU; vitamin E, 60 IU; cobalamin, 23 μg; riboflavin, 5.5 mg; niacin, 30 mg; pantothenic acid, 8 mg; choline, 500 mg; menadione, 1.2 mg; folic acid, 0.9 mg; pyridoxine, 1.2 mg; thiamine, 1.7 mg; biotin, 55 μg; manganese, 90 mg; zinc, 86 mg; iron, 55 mg; copper, 5.5 mg; iodine, 1.6 mg; selenium, 0.3 mg.

**Table 3:** Effects of energy-restricted feeding and switching to ad libitum feeding on ADG (g/bird per day), ADFI (g/bird per day), FCR (g/g), and mortality (%) of the pullets from 8 to 21 weeks of age. ^1.^

	Control	Energy-Restricted Feeding (8–18 Week)					*p*-Value		
AME_n_, kcal/kg	2850	2750	2650	2550	2450	SEM ^2^	Energy Restriction Level	Linear	Quadratic
8–13 weeks									
ADG	8.06 ^a^	7.50 ^b^	7.08 ^c^	6.75 ^d^	6.26 ^e^	0.106	<0.001	<0.001	0.494
ADFI	43.14	43.14	43.14	43.15	43.14	0.003	0.920	0.877	0.575
FCR	5.36 ^e^	5.76 ^d^	6.10^c^	6.40 ^b^	6.90 ^a^	0.091	<0.001	<0.001	0.579
13–18 weeks									
ADG	8.44 ^a^	8.21 ^a^	7.90 ^b^	7.58 ^c^	7.54 ^c^	0.069	<0.001	<0.001	0.333
ADFI	52.12	52.12	52.12	52.12	52.12	0.000	0.421	1.000	0.240
FCR	6.18 ^c^	6.35 ^c^	6.61 ^b^	6.89 ^a^	6.93 ^a^	0.058	<0.001	<0.001	0.451
8–18 weeks									
ADG	8.25 ^a^	7.86 ^b^	7.49 ^c^	7.16 ^d^	6.90 ^e^	0.081	<0.001	<0.001	0.206
ADFI	47.62	47.63	47.63	47.64	47.63	0.007	0.920	0.464	0.591
FCR	5.78 ^e^	6.06 ^d^	6.36 ^c^	6.66 ^b^	6.91 ^a^	0.068	<0.001	<0.001	0.689
18–21 weeks									
ADG	5.77 ^c^	6.91 ^b^	7.32 ^b^	8.49 ^a^	9.24 ^a^	0.239	<0.001	<0.001	0.988
ADFI	64.55 ^b^	66.50 ^b^	67.23 ^b^	66.75 ^b^	71.58 ^a^	0.550	<0.001	<0.001	0.214
FCR	11.47 ^a^	9.67 ^b^	9.41 ^b^	7.98^c^	7.77 ^c^	0.285	<0.001	<0.001	0.238
8–21 weeks									
ADG	7.67	7.64	7.45	7.47	7.44	0.041	0.180	0.027	0.515
ADFI	51.53 ^b^	51.99 ^b^	52.15 ^b^	52.05 ^b^	53.16 ^a^	0.128	<0.001	<0.001	0.227
FCR	6.72 ^c^	6.81 ^bc^	7.01 ^ab^	6.98 ^ab^	7.15 ^a^	0.042	0.006	<0.001	0.828
Mortality	0.83	0.83	0.83	0.83	0.42	0.223	0.972	0.617	0.672

^1^ Values are means of eight replicates per dietary treatment. ^2^ SEM, Standard Error of Mean (The same as below). ^a–e^ Means without a common superscript within a row differ significantly (*p* < 0.05). AMEn, apparent metabolizable energy corrected for nitrogen.

**Table 4:** Effects of energy-restricted feeding and switching to ad libitum feeding on ADEI (kcal AME_n_/day) and ECR (kcal AME_n_/ g) of pullets from 8 to 21 weeks of age. ^1.^

	Control	Energy-Restricted Feeding (8–18 Week)					*p*-Value		
AME_n_, kcal/kg	2850	2750	2650	2550	2450	SEM	Energy Restriction Level	Linear	Quadratic
8–13 weeks									
ADEI ^2^	122.87 ^a^	118.64 ^b^	114.33 ^c^	110.03 ^d^	105.70 ^e^	0.973	<0.001	<0.001	0.593
ECR ^3^	15.27 ^c^	15.84 ^b^	16.15 ^b^	16.32 ^b^	16.90 ^a^	0.121	<0.001	<0.001	0.862
13–18 weeks									
ADEI	148.55 ^a^	143.33 ^b^	138.12 ^c^	132.91 ^d^	127.69 ^e^	1.180	<0.001	<0.001	0.810
ECR	17.62	17.46	17.50	17.57	16.97	0.133	0.217	0.086	0.288
8–18 weeks									
ADEI	135.71 ^a^	130.99 ^b^	126.22 ^c^	121.47 ^d^	116.70 ^e^	1.076	<0.001	<0.001	0.646
ECR	16.46 ^b^	16.67 ^ab^	16.86 ^ab^	16.98 ^a^	16.92 ^a^	0.065	0.035	0.008	0.254
18–21 weeks									
ADEI	177.51 ^c^	182.88 ^b^	184.89 ^b^	183.56 ^b^	196.83 ^a^	1.512	<0.001	<0.001	0.214
ECR	31.53 ^a^	26.59 ^b^	25.88 ^b^	21.95 ^c^	21.37 ^c^	0.784	<0.001	<0.001	0.238
8–21 weeks									
ADEI	145.35 ^a^	142.97 ^b^	139.76 ^c^	135.80 ^d^	135.19 ^d^	0.684	<0.001	<0.001	0.230
ECR	18.97 ^a^	18.72 ^ab^	18.79 ^ab^	18.20 ^b^	18.18 ^b^	0.106	0.046	0.005	0.797

^1^ Values are means of eight replicates per dietary treatment. ^2^ Calculated energy intake (kcal AMEn/d). Calculated AMEn values of the diets are presented in Table 1. ^a–e^ Means without a common superscript within a row differ significantly (*p* < 0.05). ^3^ ECR, expressed as kcal calculated AMEn per g BW gain. AMEn, apparent metabolizable energy corrected for nitrogen.

**Table 5:** Effects of energy-restricted feeding and switching to ad libitum feeding on tarsus development of the pullets at 18 and 21 weeks of age. ^1.^

AME_n_, kcal/kg	Control	Energy-Restricted Feeding (8–18 Week)					*p*-Value		
	2850	2750	2650	2550	2450	SEM	Energy Restriction Level	Linear	Quadratic
18 weeks									
Absolute tarsus length (cm)	7.65	7.64	7.66	7.67	7.65	0.008	0.835	0.661	0.616
Absolute tarsus circumference (cm)	3.32 ^a^	3.30 ^ab^	3.30 ^ab^	3.28 ^b^	3.28 ^b^	0.005	0.039	0.005	0.313
Relative tarsus length (cm/kg)	7.82 ^c^	8.02 ^b^	8.12 ^b^	8.39 ^a^	8.45 ^a^	0.019	<0.001	<0.001	0.400
Relative tarsus circumference (cm/kg)	3.40 ^c^	3.46 ^b^	3.50 ^b^	3.58 ^a^	3.63 ^a^	0.009	<0.001	<0.001	0.920
21 weeks									
Absolute tarsus length (cm)	7.71	7.83	7.73	7.84	7.59	0.047	0.421	0.487	0.181
Absolute tarsus circumference (cm)	3.58	3.46	3.50	3.49	3.38	0.030	0.346	0.086	0.843
Relative tarsus length (cm/kg)	5.99	6.12	6.04	6.07	6.38	0.086	0.146	0.027	0.844
Relative tarsus circumference (cm/kg)	2.69	2.70	2.73	2.70	2.84	0.039	0.584	0.152	0.770

^1^ Values are means of eight replicates per dietary treatment. ^a–c^ Means without a common superscript within a row differ significantly (*p* < 0.05). AMEn, apparent metabolizable energy corrected for nitrogen.

**Table 6:** Effects of energy-restricted feeding and switching to ad libitum feeding on carcass and visceral organ development (%) of the pullets at 18 and 21 weeks of age. ^1.^

	Control	Energy-Restricted Feeding (8–18 Week)					*p*-Value		
AME_n_, kcal/kg	2850	2750	2650	2550	2450	SEM	Energy Restriction Level	Linear	Quadratic
18 weeks									
Breast index	11.18	11.26	11.46	11.31	12.03	0.139	0.319	0.081	0.423
Thigh index	15.50	15.66	16.56	15.90	16.46	0.200	0.354	0.133	0.655
Abdominal fat index	3.38 ^a^	2.23 ^ab^	2.61 ^ab^	2.17 ^ab^	1.62 ^b^	0.199	0.043	0.010	0.812
Head index	3.04	3.20	3.16	3.38	3.25	0.040	0.096	0.031	0.316
Wing index	8.52	8.78	8.74	8.80	8.48	0.125	0.893	0.947	0.336
Foot index	2.73	2.72	2.82	2.77	2.73	0.047	0.956	0.875	0.575
Heart index	0.34	0.34	0.33	0.32	0.35	0.005	0.395	0.922	0.151
Liver index	1.62 ^ab^	1.43 ^b^	1.57 ^b^	1.60 ^b^	1.94 ^a^	0.054	0.041	0.030	0.028
Spleen index	0.13	0.13	0.12	0.12	0.13	0.004	0.899	0.641	0.599
Lung index	0.44	0.47	0.40	0.40	0.41	0.012	0.231	0.127	0.781
Kidney index	0.41	0.43	0.42	0.45	0.46	0.014	0.737	0.190	0.966
21 weeks									
Breast index	12.05	12.51	11.97	12.30	11.79	0.195	0.804	0.606	0.526
Thigh index	15.53	15.89	15.93	15.82	15.61	0.194	0.962	0.945	0.456
Abdominal fat index	4.52	3.83	4.13	3.27	3.30	0.220	0.319	0.060	0.884
Head index	2.63	2.81	2.72	2.78	2.89	0.041	0.353	0.098	0.957
Wing index	2.52	2.54	2.49	2.55	2.55	0.033	0.975	0.737	0.803
Foot index	7.96	8.48	7.97	8.26	7.88	0.120	0.485	0.654	0.329
Heart index	0.31	0.33	0.33	0.33	0.35	0.006	0.583	0.129	0.926
Liver index	1.67	1.75	1.49	1.66	1.87	0.058	0.333	0.463	0.159
Spleen index	0.09	0.11	0.11	0.12	0.11	0.005	0.293	0.106	0.324
Lung index	0.34	0.37	0.37	0.37	0.41	0.011	0.497	0.119	0.836
Kidney index	0.43	0.47	0.45	0.48	0.50	0.014	0.580	0.157	0.983

^1^ Values are means of eight replicates per dietary treatment. ^a,b^ Means without a common superscript within a row differ significantly (*p* < 0.05). AMEn, apparent metabolizable energy corrected for nitrogen.

**Table 7:** Effects of energy-restricted feeding and switching to ad libitum feeding on PP traits and the relative weights of the GIT (g/kg BW) of the pullets at 18 and 21 weeks of age. ^1.^

	Control	Energy-Restricted Feeding (8–18 Week)					*p*-Value		
AME_n_, kcal/kg	2850	2750	2650	2550	2450	SEM	Energy Restriction Level	Linear	Quadratic
18 weeks									
PP amount (n)	63.88	66.38	67.88	62.88	71.00	1.453	0.421	0.302	0.698
PP amount index (n/ kg)	58.03	62.34	64.26	62.38	69.53	1.607	0.066	0.046	0.888
Relative weight (g/ kg)									
Proventriculus	2.41	2.61	2.65	2.50	2.66	0.057	0.610	0.355	0.595
Gizzard	16.19	16.73	15.48	17.89	17.46	0.357	0.208	0.139	0.553
Small intestine ^2^	27.32 ^c^	27.87 ^c^	27.91 ^c^	28.94 ^b^	31.67 ^a^	0.258	<0.001	<0.001	0.293
Duodenum	6.58 ^b^	6.84 ^ab^	6.70 ^ab^	6.93 ^ab^	7.26 ^a^	0.103	0.009	0.012	0.877
Jejunum	11.39 ^b^	11.63 ^ab^	11.60 ^ab^	11.87 ^ab^	12.20 ^a^	0.122	<0.001	0.007	0.687
Ileum	9.35 ^b^	9.40 ^b^	9.61 ^b^	10.14 ^a^	10.33 ^a^	0.089	0.016	0.012	0.534
Caeca	4.13 ^c^	4.32 ^c^	4.29 ^b^	4.56 ^a^	4.80 ^a^	0.200	0.028	0.040	0.642
Rectum	2.94	2.98	2.68	3.07	3.19	0.065	0.097	0.108	0.256
21 weeks									
PP amount (n)	68.29	71.88	69.75	72.38	74.13	1.522	0.799	0.917	0.782
PP amount index (n/kg)	53.97	56.39	54.15	55.98	62.78	1.583	0.211	0.617	0.480
Relative weight (g/kg)									
Proventriculus	2.53	2.44	2.41	2.67	2.74	0.075	0.569	0.217	0.372
Gizzard	13.70	14.33	14.45	14.78	14.05	0.407	0.799	0.473	0.319
Small intestine ^2^	15.25 ^d^	15.80 ^c^	16.34 ^ab^	16.59 ^ab^	16.96 ^a^	0.197	<0.001	<0.001	0.587
Duodenum	4.02 ^b^	4.23 ^ab^	4.21 ^ab^	4.39 ^a^	4.58 ^a^	0.202	0.014	0.016	0.642
Jejunum	6.44 ^b^	6.59 ^b^	6.87 ^ab^	7.03 ^ab^	7.25 ^a^	0.107	<0.001	0.011	0.782
Ileum	4.79	4.98	5.26	5.17	5.13	0.223	0.261	0.109	0.658
Caeca	3.17	3.24	3.42	3.44	3.41	0.156	0.265	0.092	0.324
Rectum	2.05	2.12	2.07	2.26	2.25	0.078	0.190	0.126	0.129

^1^ Values are means of eight replicates per dietary treatment. ^2^ Small intestine = duodenum + jejunum + ileum. ^a–d^ Means without a common superscript within a row differ significantly (*p* < 0.05). AMEn, apparent metabolizable energy corrected for nitrogen.

**Table 8:** Effects of energy-restricted feeding and switching to ad libitum feeding on the relative length of the GIT (cm/kg BW) of the pullets at 18 and 21 weeks of age. ^1.^

	Control	Energy-Restricted Feeding (8–18 Weeks)					*p*-Value		
AME_n_, kcal/kg	2850	2750	2650	2550	2450	SEM	Energy Restriction Level	Linear	Quadratic
18 weeks									
Small intestine ^2^	105.60 ^c^	106.32 ^bc^	106.34 ^b^	106.93 ^a^	107.73 ^a^	1.648	<0.001	<0.001	0.798
Duodenum	20.75 ^b^	20.94 ^ab^	21.00 ^ab^	21.21 ^a^	21.45 ^a^	0.691	0.011	0.003	0.625
Jejunum	43.10 ^b^	43.47 ^ab^	43.33 ^ab^	43.64 ^a^	43.95 ^a^	1.042	0.047	0.032	0.905
Ileum	41.75 ^c^	41.91 ^bc^	42.01 ^b^	42.08 ^ab^	42.33 ^a^	0.891	0.031	0.029	0.872
Caeca	12.22 ^c^	12.52 ^bc^	12.69 ^bc^	12.59 ^bc^	13.07 ^a^	0.452	0.025	0.040	0.426
Rectum	7.17	7.25	7.20	7.26	7.53	0.219	0.105	0.197	0.357
21 weeks									
Small intestine ^2^	102.53 ^c^	103.67 ^b^	103.16 ^b^	103.66 ^b^	104.38 ^a^	2.312	0.011	0.009	0.371
Duodenum	20.19 ^b^	20.35 ^b^	20.27 ^b^	20.69 ^ab^	21.08 ^a^	1.001	0.004	0.028	0.849
Jejunum	41.29 ^b^	41.53 ^b^	41.81 ^ab^	41.73 ^ab^	42.08 ^a^	0.980	0.013	0.032	0.613
Ileum	41.05	41.79	41.08	41.24	41.22	0.887	0.306	0.289	0.719
Caeca	12.06	12.32	12.30	12.57	13.07	0.303	0.089	0.045	0.381
Rectum	7.39	7.24	7.14	7.19	7.26	0.201	0.229	0.135	0.339

^1^ Values are means of eight replicates per dietary treatment. ^2^ Small intestine = duodenum + jejunum + ileum. ^a–c^ Means without a common superscript within a row differ significantly (*p* < 0.05). AMEn, apparent metabolizable energy corrected for nitrogen.

## Data Availability

Not applicable.
